# The first Pleistocene fossil records of *Urtica kioviensis* Rogow. (Urticaceae) and *Potamogeton sukaczevii* Wieliczk. (Potamogetonaceae) in the British Isles

**DOI:** 10.1007/s00334-018-0679-6

**Published:** 2018-05-30

**Authors:** Michael H. Field, Simon G. Lewis

**Affiliations:** 1 Faculty of Archaeology, Leiden University, van Steenisgebouw, Einsteinweg 2, 2333CC Leiden, The Netherlands; 2 School of Geography, Queen Mary University of London, Mile End Road, London, E1 4NS UK

**Keywords:** *Urtica kioviensis*, *Potamogeton sukaczevii*, Ipswichian Stage, Palaeoclimate, Late Pleistocene, UK

## Abstract

Seeds of the extant *Urtica kioviensis* Rogow. (Urticaceae) and endocarps of the extinct *Potamogeton sukaczevii* Wieliczk. (Potamogetonaceae) were recorded in diverse plant macrofossil assemblages recovered from organic sediments exposed during excavations at Saham Toney, Norfolk, UK. Aminostratigraphical data show the sediments were deposited during the Ipswichian (Last Interglacial) Stage. Palynological data indicates deposition during the *Carpinus* pollen zone of the Ipswichian Stage—the latter part of pollen zone Ip IIb and Ip III. The records are noteworthy not only because they are the first in the British Pleistocene but also because of the geographical occurrences of these two species. *Urtica kioviensis* is absent from the British flora today and has a modern range in central and eastern Europe (only extending as far west as north–east Germany and Denmark), while the extinct *Potamogeton sukaczevii* has only been recovered from Late Pleistocene sediments in Belarus, Lithuania, Poland and western Russia. The presence of *U. kioviensis* along with other exotic species to the British Isles (e.g. *Najas minor* L. and *Salvinia natans* L., which today have central and southern ranges in Europe and in the case of *S. natans* occurs on other continents) may point to more continental conditions or warmer summer conditions during the second half of the Ipswichian Stage in southern Britain. No modern analogues occur in Britain for the assemblages recovered from Saham Toney. Evidence of colder winters or at least warmer summers at the time of deposition does not support the view that sea-level peaked in the *Carpinus* zone of the Eemian Stage (correlated with the Ipswichian Stage) associated with increased oceanicity. Southern Britain would have been under the influence of the Atlantic Ocean and a degree of oceanicity is supported by the presence of two thermophilous taxa, *Hedera* and *Ilex*, in the pollen spectra from Saham Toney. Alternative explanations for the presence of these exotic species are that they were tolerating mild winters and cooler summers at the time of deposition or exploiting suitable micro-environments. The distribution of *P. sukaczevii* is probably an artefact of the distribution of expertise in the identification of *Potamogeton* fossil endocarps rather than having any palaeogeographic or palaeoclimatic significance. It is an extinct ancestor of the extant *P. maackianus* A. Benn, an eastern Asian pondweed. Its discovery in Britain encourages a reassessment of plant macrofossil assemblages from western Europe, which may lead to a consideration of the relationship between the Late Pleistocene vegetation of Europe and eastern Asia.

## Introduction

**Fig. 1:**
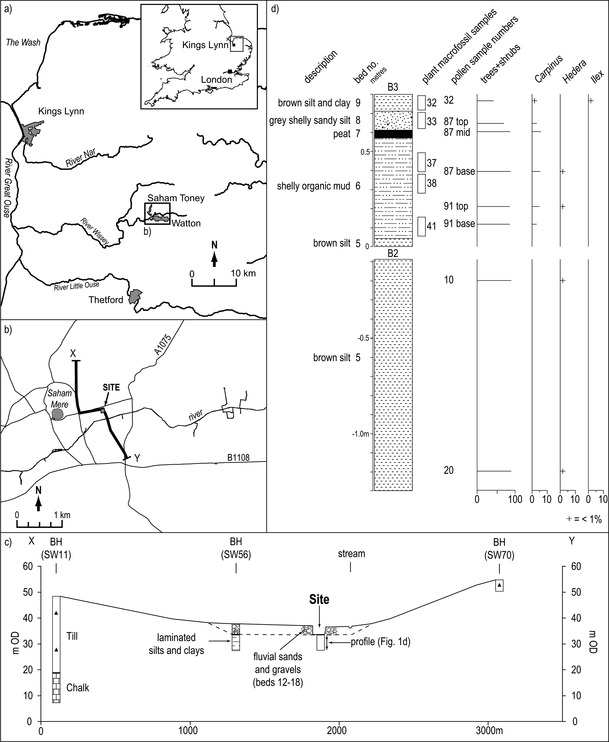
**a** Western Norfolk; **b** The location of the site and cross section X–Y; **c** Cross section X–Y through the site including British Geological Survey borehole records (contains BGS materials, NERC 2016); **d** Summary stratigraphic log of beds 5–9 (overlying deposits, including fluvial sands and gravels are not shown) with the positions of the plant macrofossil and pollen samples (top, mid and base refers to position of subsamples within monoliths) and summary palynological data. B2 and B3 are adjacent sections, but shown in stratigraphic position

Investigations into the Pleistocene deposits at Saham Toney, Norfolk (52°35′56″N, 0°49′17″E; NGR TF 9125, 0197) were undertaken in 2007 during the construction of an artificial fishing lake. Samples collected from this site have yielded a range of palaeoenvironmental information, including plant macrofossils of two angiosperm species not hitherto known from the British Pleistocene record: *Urtica kioviensis* Rogow. (Urticaceae) and *Potamogeton sukaczevii* Wieliczk. (Potamogetonaceae). The site is some 33 km west–south–west of Norwich, within a tributary of the river Wissey, which drains a catchment in central Norfolk and flows westward into the Wash basin (Fig. 1). The pre-Pleistocene geology of the area is Cretaceous Chalk and the Pleistocene deposits locally consist of glaciogenic sediments of the Anglian Stage (MIS 12) Lowestoft Formation and, along the course of the rivers, post-Anglian Stage fluvial sands and gravels and Holocene alluvium (Fig. 1). Excavation of these sands and gravels to construct the fishing lake resulted in recovery of fragments of mammoth teeth from the basal gravels and the exposure of underlying organic sediments. A series of trenches, sections and boreholes enabled the stratigraphy at the site to be established and the deposits to be sampled for palaeoenvironmental analysis.

This paper provides a summary of the site stratigraphy and describes the new plant macrofossil records in advance of a full report on the multi-disciplinary investigations of this locality.

The lithostratigraphy comprises up to 6 m of the organic sediments (beds 3–11), these underlie medium to coarse gravels with minor sand, clay and peat facies (beds 12–18) which were laid down under cold climate conditions during the Devensian Stage (Weichselian Stage) (Fig. 1c, d). The sedimentology of the organic sediments, which are predominantly fine-grained, indicates deposition in slow-flowing to standing water with some terrestrial peat accumulation. Aminostratigraphical data from *Bithynia* opercula contained within the organic sediments (Penkman et al. 2011) dates them to the Last Interglacial Stage (Ipswichian Stage in Britain, Eemian Stage in continental Europe and MIS 5e in the deep ocean record). The palynological information, together with molluscan and coleopteran assemblages, indicates that full interglacial conditions developed during their deposition. The pollen spectra from these sediments indicate the initial establishment of boreal woodland, followed by deciduous *Quercus*/*Corylus* woodland, with later arrival of *Carpinus*. Biostratigraphically, this vegetation pattern is typical of the Ipswichian Stage and in particular pollen zones Ip I–Ip III (West 1980).

The pollen spectra of beds 6–8 (Fig. 1d) show a dominance of trees and shrubs including *Carpinus*. It is not present in the underlying brown silt (bed 5), but increases to levels between 5–10% in beds 6–8, while in bed 9 *Carpinus* is only present in small quantities (< 1%). *Hedera* is present in beds 5 and 6, while *Ilex* is recorded in bed 9. The latter two thermophilous taxa have oceanic preferences as they do not tolerate cold winters (Iversen 1944). The pollen assemblages recovered from beds 6, 8 and 9 allow correlation with the latter part of Ipswichian Stage pollen zone Ip IIb or the beginning of Ip III.

## The new plant records

Twenty-five sediment samples were collected from Saham Toney for plant macrofossil analysis. All plant macrofossils present in 200 cm^3^ of sediment from each sample were picked out to produce concentration data. The samples yielded diverse plant macrofossil assemblages. Five samples contained remains of *U. kioviensis* and *P. sukaczevii*. These samples are from beds 6, 8 and 9; the shelly organic mud (samples 41, 38 and 37), grey shelly sandy silt (sample 33) and brown silt and clay (sample 32). Plant macrofossil data from these sediment samples show that the water body was surrounded by damp woodland which included *Alnus glutinosa*. Where the drainage was better, *Betula* and *Taxus baccata* grew. Reed swamp occupied the margins of the water body which included taller plants such as *Schoenoplectus lacustris, Sparganium erectum* and *Typha*. Where enough light penetrated, shorter plants such as *Lycopus europaeus, Mentha* cf. *aquatica* and *Solanum dulcamara* grew in the reed swamp. The presence of *Cyperus fuscus* and *Lythrum portula* suggests that muddy areas also could be found at the water’s edge. The water body contained a diverse flora of floating (e.g. *Lemna* and *Salvinia natans*), emergent (e.g. *Potamogeton crispus* and *P. natans*) and submergent (e.g. *Ceratophyllum demersum, Najas flexilis, N. minor* and *N. marina*) plants. The aquatic component of the plant macrofossil assemblages indicates basic, mesotrophic conditions in still or slow-moving fresh water that contained little suspended sediment.

## The *Urtica kioviensis* Rogow. fossil seeds

**Fig. 2:**
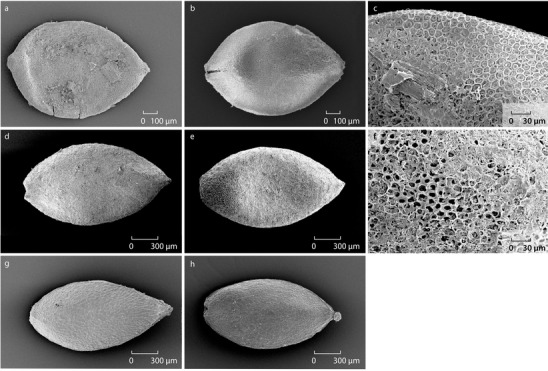
**a, b***Urtica dioica* fossil seeds from Saham Toney (sample 33); **c** the surface cells on a fossil specimen of a *U. dioica* from Saham Toney (sample 33); **d, e***U. kioviensis* fossil seeds from Saham Toney (sample 32); **f** the surface cells on a fossil specimen of an *U. kioviensis* from Saham Toney (sample 32); **g, h** modern achenes of *U. kioviensis* collected by E. Pechenyuk on the 19/9/1995 on the banks of the river Savala, Novokhopiorsk, Russia (these specimens were kindly given to Field by E. Pechenyuk in 2000)

*Urtica kioviensis* is monoecious and produces, after fertilization, achenes (dry, one-seeded, indehiscent fruits). The fruit wall is termed the pericarp if the fruit develops from a single ovary as in the case of *U. kioviensis*. The pericarp has not preserved on the fossil specimens from Saham Toney and, thus, the specimens are called seeds. Samples 32 (7 seeds), 37 (4 seeds) and 38 (4 seeds) yielded seeds of *U. kioviensis*. The fossil seeds are ovoid and appear flat but are slightly biconvex in side view (Fig. 2d, e). The greatest width is in the centre of the seed. At the base is a broad scar, while the apex tapers. The length of the Saham Toney seeds ranges between 1.85 and 1.58 mm, while the width ranges between 0.93 and 0.87 mm. Wolters et al. (2005) present additional biometric data for comparison. The fossil specimens are shown (Fig. 2d, e) alongside modern material (Fig. 2g, h) for comparison of their shape. The modern specimens shown in Fig. 2 are achenes because the seed is still encased in the pericarp. It was difficult to remove the pericarps from the modern achenes as they closely adhere to the seeds. At the tip of the achene is a persistent, withered brush-like stigma. This feature has not preserved in the fossil specimens from Saham Toney.

The plant macrofossil assemblages also include seeds of the dioecious *U. dioica* (Fig. 2a, b). The clear size and morphological differences between the seeds of *U. kioviensis* and *U. dioica* from Saham Toney are illustrated in Fig. 2. Wolters et al. (2005) provide a useful morphological comparison of the fruits of these two species as well as others in the genus *Urtica*.

## The *Potamogeton sukaczevii* Wieliczk. fossil endocarps

Preston (1995) comments that many terms have been used for modern propagules from species in the genus *Potamogeton* including drupe, drupelet, achene, nut and nutlet. He concluded that none of these are entirely satisfactory and preferred the term fruit. However, in a fossil context it is often the case that not all the fruit is preserved. Aalto (1970) recognized that the outer part of the fruit wall (the pericarp) is composed of exocarp cells below which is found a thin fleshy layer (parenchymatous mesocarp). Often these do not preserve. What remains is the sclerified mesocarp sometimes termed the fruit stone, but also called the endocarp. Here, Aalto’s example is followed and the term endocarp is applied to the fossils.

**Fig. 3:**
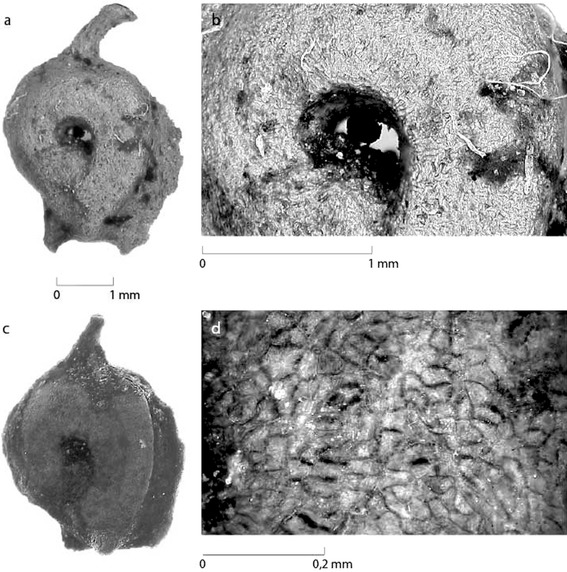
**a, b** Fossil *Potamogeton sukaczevii* endocarp kindly given to Field by Velichkevich in 1998 (from Gołków, Poland: sample Q-11/64 A) and a close up of its surface cells; **c, d** Fossil *P. sukaczevii* endocarp from Saham Toney (sample 41) and a close-up of its surface cells

At Saham Toney, samples 41 (2 endocarps) and 33 (13 endocarps) yielded endocarps of *P. sukaczevii*. Figure 3c, d shows a fossil *P. sukaczevii* endocarp from sample 41 and, for comparison, a fossil *P. sukaczevii* endocarp kindly given to M. H. Field by Velichkevich in 1998 (from Gołków, Poland; sample Q-11/64 A) is shown in Fig. 3a, b. The endocarps from Saham Toney are between 2.75 and 2.98 mm long and 2.25 and 2.86 mm wide. The length measurement was taken from the base of the style to the base of the endocarp. Velichkevich and Zastawniak (2006) describe the endocarps as broadly obovate in outline but it is clear in Fig. 3 that the endocarps are asymmetrical. Like the Polish specimen in Fig. 3a and those illustrated in Velichkevich and Zastawniak (2006), the Saham Toney specimens have an irregularly sigmoid ventral margin (the side opposite to the one where the lid is positioned). The upper two-thirds are very convex while at the base is a straight section near the stalk. The lid, on the dorsal margin, is slightly curved, keeled and terminates just short of the style base in a point. Velichkevich and Zastawniak (2006) comment that eastern European *P. sukaczevii* endocarps often possess a wart at the base of the lid. This was not evident in the Saham Toney specimens, but it may be that the base of the lid has been truncated (see Fig. 3c). The style is in a central position and slightly tilted towards the dorsal side. The Saham Toney endocarps have a large stalk, flat sides with a small but deep central depression in the shape of a comma whose tail points towards the stalk, an inconspicuous furrow running parallel to the dorsal margin along most of its length and pronounced, large mamillate warts at the base of both sides of the endocarp—all features consistent with Velichkevich and Zastawniak’s (2006) description of the endocarp morphology of *P. sukaczevii*. As with the eastern European fossils, the Saham Toney endocarps have, in places, some of the fleshy mesocarp still adhering to the endocarp. The sclerified mesocarp cells on the surface of the endocarp are very characteristic (Fig. 3b, d).

## Implications of these new records

This paper reports on the first records of *U. kioviensis* and *P. sukaczevii* in the British Pleistocene. The Saham Toney organic sediments have been correlated with the Ipswichian Stage on aminostratigraphical, biostratigraphical and lithostratigraphical grounds. *Carpinus* is present in beds 6–8, which indicates sediment deposition during the middle and latter half of the Last Interglacial Stage (either the latter part of pollen zone Ip IIb or Ip III). The presence of *P. sukaczevii*, an extinct freshwater pondweed, which is a characteristic component of Eemian plant macrofossil assemblages recovered from Belarus, Lithuania, Poland and western Russia (Velichkevich and Granoszewski 1996; Granoszewski 2003; Velichkevich et al. 2005; Velichkevich and Zastawniak 2006), is also consistent with a Last Interglacial Stage age. It should be noted that stratigraphically *P. sukaczevii* also occurs not only in the Eemian Stage but occasionally in the early Vistulian Stage, both in an interstadial and during the glacial periods, such as at Horoszki Duże, Poland (Granoszewski 2003). The Vistulian Stage is correlated with the Devensian Stage in Britain and the Weichselian Stage in western Europe.

Compared to the Holocene, the climate of the Eemian Stage was generally more oceanic in western and central Europe (Zagwijn 1996; Aalbersberg and Litt 1998). At the beginning of the Eemian Stage, differences between summer and winter temperatures were at a maximum and Zagwijn (1996) interpreted the early Eemian finds of thermophilous trees and aquatic plants as indicators of continental conditions with warmer summers. However, 2,000–3,000 years into the Last Interglacial Stage there was a change to a more oceanic climate, which persisted throughout the rest of the stage. Using the indicator species method Zagwijn (1996) reconstructed winter temperatures at Amsterdam to have been at a high of 3 °C in the *Carpinus* pollen zone (E5). He noted that the winter temperature trend throughout the stage matched that of the rise and fall of sea-level, so that oceanicity during the stage reflected transgression and regression of the sea. Sea-level reached its peak during the climatic optimum of the Eemian Stage (the *Carpinus* pollen zone E5) and oceanic conditions prevailed (Zagwijn 1989). Palynological data from beds 6 and 9 at Saham Toney support the conclusion that during the *Carpinus*-phase conditions were oceanic in southern England with the presence of *Hedera* and *Ilex*, two taxa that Iversen (1944) showed were not tolerant of cold winters. This is unsurprising owing to the proximity of the Atlantic Ocean.

**Fig. 4:**
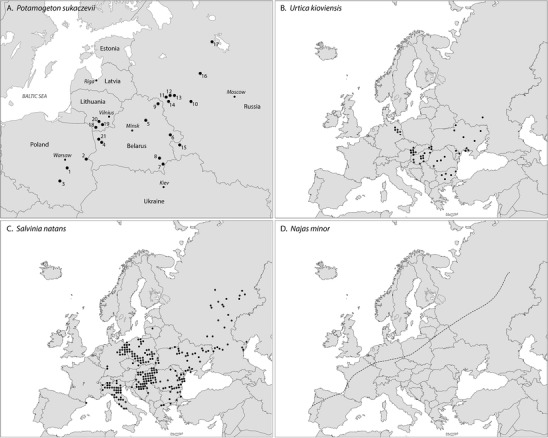
**a** The distribution of fossil sites that have yielded endocarps of *P. sukaczevii* (the position of each site is taken from Velichkevich and Zastawniak (2006) except for the positions of the Polish sites which are placed according to Granoszewski (2003). The sites are: 1—Gołków, Poland, 2—Horoszki Duże, Poland, 3—Bedlno, Poland, 4—Knyazhevodtsy, Belarus, 5—Murava, Belarus, 6—Cherikov, Belarus, 7—Loev (Loyev), Belarus, 8—Borkhov Rov, Belarus, 9—Cherny Bereg, Belarus, 10—Zabolot’e, Russia, 11—Panfilovo, Russia, 12—Koz’ya, Russia, 13—Ryasna, Russia, 14—Nizhnyaya Boyarshchina, Russia, 15—Zharki, Russia, 16—Staroe Zarech’e, Russia, 17—Shenskoe, Russia, 18—Liškyava (Lishkyava), Lithuania, 19—Yanionis (Yanyonis), Lithuania, 20—Netiesos (Nyatesos), Lithuania, 21—Komotovo, Belarus; **b** The modern distribution of *U. kioviensis* (Jalas and Suominen 1988); **c** The modern distribution of *Salvinia natans* in Europe (Jalas and Suominen 1987). Mabberley (1997) notes that it also occurs in warm temperate or tropical areas in Africa and Asia; **d** Dashed line shows the modern northern limit of *Najas minor* (Hultén and Fries 1986), who comment that *N. minor* is not native in America and that the knowledge of its distribution is partly incomplete

Three species recorded from Saham Toney (*Najas minor, Salvinia natans* and *Urtica kiovensis*) do not occur in the UK today (Stace 2010); they all have central or southern European modern distributions (Fig. 4), with *S. natans* also occurring in warm temperate or tropical areas in Africa and Asia (Mabberley 1997). If the Last Interglacial Stage (Ipswichian or Eemian Stages) climate conditions in western and central Europe were more oceanic than in the Holocene, particularly during the climatic optimum (in the *Carpinus* pollen zone E5) then it is possible that these species were tolerating milder winters and cooler temperatures during the growing season than they do today. *Urtica kioviensis* today occupies river valleys, growing in damp areas with *Phragmites, Phalaris* and *Carex riparia* reed swamp as well as *Salix* scrub and various *Alnus* plant associations (Wollert et al. 2003; Wolters 2005). *Salvinia natans* is a floating aquatic pteridophyte that favours standing, fresh water, while *Najas minor* also lives in slow-moving fresh water where light penetrates down into the water column. Alternatively, these species may have exploited suitable micro-environments possibly being transported from further afield by wildfowl.

Until its discovery at Saham Toney *P. sukaczevii* had only been found from Late Pleistocene sites in central and eastern Europe (Fig. 4). It is probable that this distribution is an artefact of the distribution of expertise in the identification of *Potamogeton* fossil endocarps rather than having any palaeogeographic or palaeoclimatic significance. *Potamogeton sukaczevii* is an extinct species most closely related to the extant *P. maackianus* A. Benn, which today is distributed on the Korean Peninsula, in Japan, throughout the Russian Far East, China, Taiwan, Indonesia, Philippines, Myanmar and Vietnam (Ito et al. 2009; Zhang 2009). *Potamogeton sukaczevii* is the youngest species in the entire *P. maackianus* phylogenetic group (Field et al. 2000; Granoszewski 2003). Its occurrence in the British Isles during the Late Pleistocene is an opportunity to prompt a consideration of the relationships between the European and eastern Asian vegetation. However, much more work, including a reassessment of western European plant macrofossil assemblages, is needed to achieve this.

## Conclusions

The occurrence of *U. kioviensis* together with *Najas minor* and *Salvinia natans*, all of which are exotic to the UK today but occurring in southern or central positions on the European continent, from the latter part of the Last Interglacial Stage at Saham Toney add new data to determine palaeoclimatic conditions in Britain at this time. Previous palaeoclimate reconstructions for the middle and latter part of the Last Interglacial Stage in western Europe show that oceanic conditions prevailed. Therefore, these species either were exploiting more continental type microenvironments or they were tolerating warmer winters and/or cooler temperatures during the growing season than they presently do during the time of sediment deposition.

The recognition of *U. kioviensis* and *P. sukaczevii* for the first time in the British Pleistocene provides new data on the past distribution of these species. However, caution must be used in regarding these exotic or extinct taxa as rare in the British or western European Pleistocene sediments, as they may not have been recognized in previous investigations of western European Pleistocene sediments. The use of modern reference collections that include material from eastern Europe and knowledge of the eastern European palaeocarpological literature, for example the works of Dorofeev, Nikitin and Velichkevich (e.g. Nikitin 1957; Dorofeev 1977; Velichkevich 1982) is needed to become familiar with these taxa. Further work on British Late Pleistocene plant macrofossil assemblages is needed to assess the wider significance of these findings. *Potamogeton sukaczevii* has an affinity with the extant *P. maackianus* which is found in eastern Asia today. Reassessment of previous work and new studies on western European plant macrofossils may lead to a better understanding of the relationship between European and eastern Asian vegetation through time.

## References

[CR1] Aalbersberg G, Litt T (1998). Multiproxy climate reconstructions for the Eemian and Early Weichselian. J Quat Sci.

[CR2] Aalto M (1970). Potamogetonaceae fruits. I. Recent and subfossil endocarps of the Fennoscandian species. Acta Bot Fenn.

[CR3] Dorofeev PI (1977). K sistematike neogenovykh *Potamogeton* Belorussi (On the taxonomy of Neogene *Potamogeton* of Belarus, in Russian (ed)). Doklady Akademii Nauk BSSR.

[CR4] Field MH, Velichkevich FY, Andrieu-Ponel V, Woltz P (2000). Significance of two new Pleistocene plant records from western Europe. Quat Res.

[CR5] Granoszewski W (2003). Late Pleistocene vegetation history and climatic changes at Horoszki Duże, eastern Poland: a palaeobotanical study. Acta Palaeobot Suppl.

[CR6] Hultén E, Fries M (1986). Atlas of north European vascular plants—north of the Tropic of Cancer.

[CR7] Ito Y, Ohi-Toma T, Tanaka N, Murata J (2009). New or noteworthy plant collections from Myanmar (3) *Caldesia parnassifolia, Nechamandra alternifolia. Potamogeton maackianus* and *P. octandrus*. J Jpn Bot.

[CR8] Iversen J (1944). *Viscum, Hedera* and *Ilex* as climate indicators—a contribution to the study of the post-glacial temperature climate. Geologiska Föreningens i Stockholm Förhandlingar.

[CR9] Jalas J, Suominen J (1987). Atlas Florae Europaeae, distribution of vascular plants in Europe. Pteridophyta and Gymnospermae.

[CR10] Jalas J, Suominen J (1988). Atlas Florae Europaeae, distribution of vascular plants in Europe. Angiospermae (part) Salicaceae to Balanophoraceae, Polygonaceae, Chenopodiaceae to Basellaceae.

[CR11] Mabberley DJ (1997). The plant-book.

[CR12] Nikitin PA (1957). Pliotsenovye i chetvertichyne flory Voronezhskoy oblasti (The Pliocene and Quaternary floras of the Voronezh region, in Russian).

[CR13] Penkman KEH, Preece RC, Bridgland DR (2011). A chronological framework for the British Quaternary based on *Bithynia* opercula. Nature.

[CR14] Preston CD (1995) Pondweeds of Great Britain and Ireland. (Botanical Society of the British Isles Handbook 8) Botanical Society of the British Isles, London

[CR15] Stace C (2010). New flora of the British Isles.

[CR16] Velichkevich FY (1982). Pleystotsenovye flory lednikovykh oblastey Vostochno-Evropeyskoy Raviny (The Pleistocene floras of glacial areas of the east-European Plain, in Russian).

[CR17] Velichkevich FY, Granoszewski W (1996). *Potamogeton sukaczevii* Wieliczk. in the Neopleistocene floras of Poland, Belarus and Lithuania. Acta Palaeobot.

[CR19] Velichkevich FY, Mamakowa K, Stuchlik L (2005). Revision of some plant macrofossil collections from the Eemian interglacial deposits of central and western Poland. Acta Palaeobot.

[CR18] Velichkevich FY, Zastawniak E (2006). Atlas of the Pleistocene vascular plant macrofossils of Central and Eastern Europe, part 1, Pteridophytes and monocotyledons.

[CR20] West RG (1980). Pleistocene forest history in East Anglia. New Phytol.

[CR21] Wollert H, Bolbrinker P, Welk E (2003). Zum Vorkommen und soziologischen Verhalten von *Urtica kioviensis* Rogowitsch in der Mecklenburgischen Schweiz (Ostmecklenburg) sowie zur gegenwärtigen Verbreitung der Art. Botanischer Rundbrief für Mecklenburg-Vorpommern.

[CR22] Wolters S, Bittmann F, Kummer V (2005). The first subfossil records of *Urtica kioviensis* Rogow. and their consequences for palaeoecological interpretations. Veget Hist Archaeobot.

[CR23] Zagwijn WH (1989). Vegetation and climate during warmer intervals in the late Pleistocene of western and central Europe. Quat Int.

[CR24] Zagwijn WH (1996). An analysis of Eemian climate in western and central Europe. Quat Sci Rev.

[CR25] Zhang S (2009). Common wetland plants in China.

